# Defining the Supportive Care Needs and Psychological Morbidity of Patients With Functioning Versus Nonfunctioning Neuroendocrine Tumors: Protocol for a Phase 1 Trial of a Nurse-Led Online and Phone-Based Intervention

**DOI:** 10.2196/14361

**Published:** 2019-12-03

**Authors:** Lisa Guccione, Karla Gough, Allison Drosdowsky, Krista Fisher, Timothy Price, Nick Pavlakis, Mustafa Khasraw, David Wyld, David Ransom, Grace Kong, Megan Rogers, Simone Leyden, John Leyden, Michael Michael, Penelope Schofield

**Affiliations:** 1 Department of Cancer Experiences Research Peter MacCallum Cancer Center Melbourne Australia; 2 Sir Peter MacCallum Department of Oncology Faculty of Medicine, Dentistry and Health Sciences The University of Melbourne Melbourne Australia; 3 Haematology and Oncology The Queen Elizabeth Hospital South Australia Australia; 4 Department of Medical Oncology Royal North Shore Hospital New South Wales Australia; 5 Department of Medical Oncology Royal Brisbane and Women's Hospital Queensland Australia; 6 Faculty of Medicine University of Queensland Brisbane Australia; 7 Medical Oncology Fiona Stanley Hospital Western Australia Australia; 8 Department of Cancer Imaging Peter MacCallum Cancer Centre Melbourne Australia; 9 Upper Gastrointestinal Cancer Service Peter MacCallum Cancer Centre Melbourne Australia; 10 Unicorn Foundation Victoria Australia; 11 Department of Medical Oncology Peter MacCallum Cancer Centre Melbourne Australia; 12 Department of Psychology and Iverson Health Innovation Research Institute Swinburne University Melbourne Australia

**Keywords:** cancer, neuroendocrine tumors, NETs, supportive care interventions, telehealth, eHealth

## Abstract

**Background:**

Online information resources and support have been demonstrated to positively influence the well-being of people diagnosed with cancer. This has been explored in past literature for more common cancers; however, for rare cancers, such as neuroendocrine tumors (NETs), there are little to no support or resources available. Despite relatively good prognoses, the quality of life (QoL) of patients with NETs is significantly lower compared with samples of mixed cancer patients and the general population. Patients with NETs also typically report unclear and difficult pathways of disease management and treatment, given the heterogeneity of the diagnosis. There is a vital need to improve the availability of disease-specific information for this patient group and provide supportive care that is tailored to the unique needs of the NET patient population.

**Objective:**

This study described the protocol of a study aimed to better understand the outcomes and experiences of patients diagnosed with NETs and to develop and pilot test a nurse-led online and phone-based intervention that will provide tailored supportive care targeted to NET subgroups (functioning vs nonfunctioning).

**Methods:**

This is a multisite cohort with 3 phases, incorporating both quantitative and qualitative data collection. Phase 1 is a mixed methods prospective cohort study of NET patients identifying differences in patient experiences and priority of needs between NET subgroups. Phase 2 utilizes results from phase 1 to develop an online and nurse-led phone-based intervention. Phase 3 is to pilot test and evaluate the intervention’s acceptability, appropriateness, and feasibility.

**Results:**

Currently, the project is progressing through phase 1 and has completed recruitment. A total of 138 participants have been recruited to the study. To date, patient-reported outcome data from 123 participants at baseline and 87 participants at 6-month follow-up have been collected. Of these, qualitative data from semistructured interviews from 35 participants have also been obtained. Phase 2 and phase 3 of the project are yet to be completed.

**Conclusions:**

Limited research for patients with NETs suggests that QoL and patient experiences are significantly impaired compared with the general population. Furthermore, past research has failed to delineate how the clinical variability between those with functioning and nonfunctioning NETs impacts patient supportive care needs. This study will improve on the availability of disease-specific information as well as informing the design of a nurse-led online and phone-based supportive care intervention tailored for the unique needs of the NET patient population.

**International Registered Report Identifier (IRRID):**

DERR1-10.2196/14361

## Introduction

### Background

It is well established that online information resources and support have a significant impact on the well-being of people diagnosed with cancer [1-3]. To date, there are several tools available for more common cancers, such as breast and prostate cancer, which have a significant impact on the patient experiences of those users [4]. Some of these tools include Internet Cancer Support Groups [5], Comprehensive Health Enhancement Support Systems [6], and bulletin boards [1,7]. More specifically, online information resources and supports were shown to increase hope, universality [1], positive emotions [7], and psychological well-being [5]. However, for those diagnosed with a rare cancer, such as neuroendocrine tumors (NETs), where patients commonly report poorer quality of life (QoL), there are little to no support or resources available [8]. This is even further complicated given that NETs can appear almost anywhere in the body [9]. In addition, NETs can range from being hormone expressive (functioning) and highly symptomatic to nonexpressive (nonfunctioning) and potentially having no symptoms at all [10]. There is an urgent need to better understand the differences in the patient experiences and information and supportive care needs of each NET subgroup (functioning vs nonfunctioning) [8]. These findings can be used to develop online information resources, tools, and support that is tailored to the 2 subtypes of NETs.

NETs are a heterogeneous group of rare cancers that derive from the neuroendocrine cell system [11]. Within Western populations, the incidence of NETs in 2012 was 14.02 per 100,000 according to the Surveillance, Epidemiology, and End Results Program [12]. Although still considered rare, the increasing prevalence of NETs is a worldwide phenomenon [12,13], possibly owing to the detection of early-stage disease and stage migration [14]. With NETs arising across the neuroendocrine system, these malignancies can affect almost any organ; however, they are most commonly found in gastrointestinal sites, typically that of the small intestine, appendix, pancreas, stomach, colon, rectum, and bronchopulmonary sites [15-18].

The clinical presentation and symptom severity of these tumors can vary greatly from patient to patient and, more broadly, between functioning and nonfunctioning NETs. NETs are classified by their secretory potential as functioning or nonfunctioning, depending on their ability to produce peptides that cause distinctive hormonal syndromes such as carcinoid syndrome [9,19,20]. Symptoms associated with functioning NETs are generally those that are caused by the secretion of hormones and those that consequently result in carcinoid syndrome or other secretory syndromes (eg, due to hypersecretion of insulin, gastrin, and glucagon). In the case of carcinoid syndrome, these symptoms largely include flushing of the skin and secretory diarrhea and are highly distressing to patients and have a significant impact on their physical and social functioning [11,21]. In addition, carcinoid syndrome can also result in clinical symptoms such as hypotension or hypertension, bronchoconstriction, and carcinoid heart disease [22]. Compared with patients with a functioning NET, patients with nonfunctioning NETs are often left managing the uncertainty of an asymptomatic cancer [23].

The 5-year survival rates for both functioning and nonfunctioning NETs are estimated at 68% [24], with treatment options varying from surgery, cytotoxic chemotherapy, somatostatin analogs, and targeted biological agents, depending on tumor location and disease stage and grade [25-29]. Despite relatively good prognoses, the QoL of patients with NETs is significantly lower compared with samples of mixed cancer patients and the general population [30-32].

#### Health-Related Quality of Life and Patient Experiences

Research on the patient experience and overall QoL of those diagnosed with NETs is limited in comparison with more common types of cancer such as breast [33], lung [34], and prostate cancer [35,36]. There is, however, evidence to suggest that QoL is significantly impaired in people with NETs when compared with the general population, as assessed by the Euopean Organization for Research and Treatment core, Quality of Life Questionnaire (EORTC QLQ-C30; Role functioning, large-sized difference, 32 points; Social functioning, medium-sized difference, 14 points; and Global QoL, large-sized difference, 18 points) [31,37]. Patients with NETs also score worse on QoL subscales compared with a mixed sample of cancer patients and survivors [30]. The severity and burden of NET-specific symptoms, for example, the frequency of bowel movements and the presence of skin flushing, have been found to correlate with a decrease in overall QoL [38]. Likewise, patients with NETs who are also experiencing symptoms associated with carcinoid syndrome report poorer QoL than patients with NETs who do not have carcinoid syndrome [30]. QoL can also be impacted by the complications of treatment of the disease itself, for example, surgery, somatostatin analogs, chemotherapy, and radiotherapy.

In addition to poorer QoL, a recent qualitative study found that patients with NETs report from their perspective that disease management and treatment pathways are unclear and difficult to navigate. It also highlighted that there is a need for support that is responsive to the specific needs of this group [8]. Indeed, patients with NETs reported low levels of satisfaction with the organization of care, and lower levels of satisfaction were associated with higher levels of anxiety and impaired psychosocial function [39]. Taken collectively, these results indicate the little to no information and support tailored to the unique needs of the NET population.

#### Principles Underpinning the Design of a Patient-Centered Intervention

To address potential differences in unmet needs between NET subgroups, the Schofield and Chambers framework will be used to develop a tailored intervention. This intervention will provide targeted supportive care for patients with NETs according to the clinical characteristics and psychosocial profile of patients with functioning and nonfunctioning NETs.

The Schofield and Chambers framework emphasizes 7 key requisite components to be considered to design and develop an intervention that will be effective, clinically feasible, and sustainable [40]. The framework builds on the Medical Research Council framework for complex interventions, which provides a guideline that can be used to assist with the development and evaluation of health interventions such as targeted supportive care and health information [41]. According to Schofield and Chambers, there are 7 key features required in the development of an intervention to achieve effective and easy translation into usual care: (1) targeting a cancer type and stage, (2) tailoring to individuals’ unique needs, (3) promoting self-management, (4) efficient intervention delivery, (5) ensuring evidence-based and theoretical grounding, (6) specifying protocol training and adherence, and (7) confirming stakeholder acceptability [40].

### Aims and Objectives

The aim of this study is to describe the protocol of the Defining NETs study. The overarching aims of this study are to better understand the outcomes and experiences of patients diagnosed with NETs and to develop and pilot test a nurse-led online and phone-based intervention that provides tailored supportive care targeted to NET subgroups.

More specifically, the objectives of this study are as follows:

1. To describe and compare the psychosocial (Patient-Reported Outcomes Measurement Information System [PROMIS] short forms assessing anxiety, depression, fatigue, pain interference, pain intensity, sleep disturbance, physical function, satisfaction with social roles and activities, supportive care needs, and experiences of the health care system), QoL (EORTC QLQ-C30 and Gastrointestinal Neuroendocrine QoL Module [GINET21]), and clinical characteristics of patients with functioning and nonfunctioning NETs within 6 months of diagnosis and then again 6 months later;
2. To conduct qualitative interviews to gain a better understanding of the experiences, care preferences, and information needs of patients with NETs;
3. To design and develop an intervention involving online triaging and the delivery of informational resources using multimedia, diagrams, and text, with phone-based nurse follow-up targeted to NET subgroups, as appropriate, and tailored to the individual’s psychosocial and clinical profile;
4. To pilot test the intervention and assess the acceptability and clinical utility of the intervention through a service that is accessible to patients nationwide from both metropolitan and rural areas.

## Methods

### Study Design

**Figure 1:**
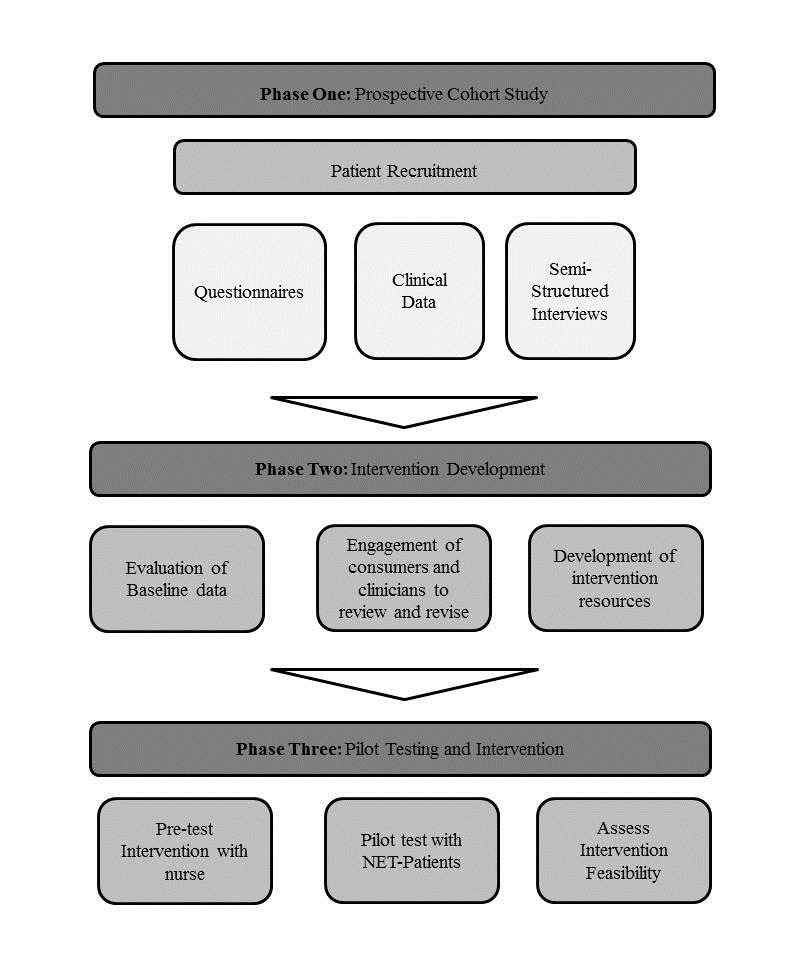
Flow chart of study phases. NET: neuroendocrine tumor.

A concurrent mixed methods triangulation design will be conducted with 3 phases incorporating both quantitative and qualitative data collection. Both data forms will be used to identify differences in patient experiences and priority of needs between the NET subgroups. Phases are displayed in Figure 1.

#### Phase 1: Prospective Cohort Study

##### Study Setting

The study will be conducted at 6 sites across Australia. Participatory sites are the Peter MacCallum Cancer Centre in Melbourne, Victoria, with additional recruitment sites at the Royal North Shore Hospital and Northern Cancer Institute (New South Wales), the Lyell McEwin Hospital (South Australia), Royal Brisbane Hospital (Queensland), and the Fiona Stanley Hospital (Western Australia).

##### Eligibility Criteria

Eligibility to this study requires a patient to (1) have a histologically confirmed diagnosis of a NET within the past 6 months, (2) be aged 18 years or older, (3) be able to understand English, (4) have their NET classified as either functioning or nonfunctioning by their treating oncologist, (5) be well enough to participate in the study as determined by the patients’ treatment team, and (6) not demonstrate psychological or cognitive difficulties that would preclude study participation as defined by the treatment team’s cognitive or psychiatric assessment or patient’s disclosed medical history.

A participant will be withdrawn if they (1) become cancer free or (2) withdraw consent. Discontinuation due to adverse events could be either at the request of the participant or the discretion of the investigator(s).

##### Outcomes and Measures

Potentially eligible patients will be identified by their treating clinician and classified as either functioning or nonfunctioning based on their clinical diagnosis. At the time of consent, patients will complete a baseline questionnaire, and thereafter a 6-month follow-up questionnaire. Profiles of the psychosocial, QoL, and clinical characteristics of patients with functioning and nonfunctioning NETs will be developed based on patient-reported outcome data and clinical data extracted from medical records at baseline and 6-month follow-up. Those participants who consent to the opt-in interview will be contacted by the study team to complete a semistructured interview.

The following measures form part of the patient questionnaires:

###### Psychological morbidity, symptoms, and functioning

- Emotional Distress Anxiety-Short Form 7a and Emotional Distress Depression-Short Form 8b
- Fatigue-Short Form 7a
- Pain Interference-Short Form 6b and Pain intensity-Short Form 3a
- Sleep Disturbance-Short Form 8b
- Physical Function-Short Form 10a
- Satisfaction with Social Roles and Activities-Short Form 6a

Emotional distress, fatigue, pain, sleep disturbance, physical functioning, and satisfaction with social functioning will be assessed using the PROMIS short forms listed above. Items comprising each short form were first evaluated using classical test theory indices. Unidimensionality was confirmed via confirmatory factor analytic techniques [42], and then, item response theory modeling and expert review were used to identify items measuring the entire spectrum of the construct targeted by each scale. All relevant short forms are standardized, accurate, and efficient self-report measures, and all these short forms were specifically developed for use in clinical oncology research [43].

###### Supportive Care Needs Survey

The Supportive Care Needs Survey (SCNS) is a 34-item questionnaire incorporating 5 scales [44]. These scales assess physical and daily living needs, psychological needs, sexuality needs, patient care and support needs, and health system and information needs. The SCNS demonstrates acceptable reliability and validity [44].

###### Cancer-Specific Health-Related Quality of Life and Gastrointestinal Neuroendocrine Module

The EORTC QLQ-C30 is a 30-item questionnaire incorporating 5 functional scales (physical, role, cognitive, emotional, and social functioning); 3 symptom scales (fatigue, pain, and nausea/vomiting), a global health status scale; and 6 single items assessing dyspnea, sleep disturbance, appetite loss, constipation, diarrhea, and financial impact [45]. Its reliability and concurrent and criterion validity have been demonstrated in numerous studies [45-47].

The QLQ-GINET21 module is a 21-item questionnaire consisting of 5 scales (endocrine, GI treatment, social function, and disease-related worries scale) and 4 single items (muscle/bone pain symptom, sexual function, information/communication function, and body image). The QLQ-GINET21 is a valid tool for assessing QoL in patients with NETs [48].

###### National Health Survey—Cancer Patient Experiences Survey

A total of 21 items have been extracted from the National Cancer Experience Survey used to assess patients’ understanding of their diagnosis and treatment, adequacy of communication, and experiences with hospital staff. These 21 items were adapted for collecting information from patients with functioning or nonfunctioning NETs about their experiences of the health care system following their diagnosis, and this survey was demonstrated to have good content validity [49].

##### Demographics and Clinical Variables

A range of demographic and clinical information will be collected pertaining to the individual’s characteristics (eg, age, gender, language, living arrangements, postcode, occupation, work status, and level of income).

Clinical data collection will occur at baseline and 6 months post baseline and will include a medical record audit to identify clinical information such as date of diagnosis, presence or absence of functional syndrome, primary site, grade, list of and frequency of diagnostic imaging, primary treatment details, length and number of hospital stays, involvement with hospital services, or referrals at the time of recruitment. At follow-up, information pertaining to the participants’ status—alive and disease free, alive with disease, lost to follow-up, or deceased (including date and cause of death and autopsy [if performed])—disease progression/recurrence (including date of detection) and progression-free survival; as well as any additional diagnostic imaging and additional treatments details will be collected.

To further investigate the supportive care needs of patients diagnosed with functioning and nonfunctioning NETs, patients will be asked to participate in an optional semistructured phone interview. Semistructured open-ended questions have been developed to allow participants to share their experiences and preferences.

##### Recruitment

Recruitment will take place from outpatient clinics and chemotherapy day units at participating sites. For each site, a research team will screen and identify new and newly diagnosed patients attending clinics that are potentially eligible. Eligibility will be confirmed with the treating clinician before any approach to clarify details from the medical records. The study will be described, and patients will be provided with a copy of the Participant Information and Consent Form (PICF), a baseline questionnaire, and a reply-paid envelope. Any questions the patient may have will be answered, and the patient will be informed that their involvement in the study is completely voluntary. If the patient wishes to participate, they will be asked to sign the PICF and complete the baseline questionnaire. Similarly, 6-month follow-up questionnaires will be posted out to participants with a reply-paid envelope for participants to return the completed questionnaires in. The study coordinator will call the patient to follow-up the return of these forms if they have not been received after a period of 1 week. Patients who decline to participate will be asked for verbal consent to collect basic demographic and clinical information from their records to examine potential recruitment bias. Reasons for refusal will be recorded.

##### Data Collection Methods

###### Patient Experience Questionnaire

All patient-reported outcome questionnaires will be collected from participants at baseline and again at 6-month follow-up in reidentifiable format and reviewed for completeness of data. Any missing data will be followed up with the participants. The reason for any remaining missing data will be noted on an electronic tracking database.

###### Medical Records Audit

All clinical data to be collected will be written into a paper-based Case Record Form, which will then be entered into an electronic database. Baseline and follow-up clinical data will be collected from the medical records of participating sites.

###### Qualitative Semistructured Interview

Patients will be asked to participate in an optional semistructured phone interview to discuss their experiences, care preferences, and information needs as a person with a NET. This interview will be conducted over the phone by a trained interviewer. On the basis of the aims, semistructured open-ended questions have been developed to allow participants to share their experiences and preferences. Given the semistructured format, additional questions and prompts will be used as necessary, as it is anticipated that themes/issues will be identified during the interview. This will allow the participant to share their individual experiences. It is anticipated that approximately 20 participant interviews will be completed for each NET group, however, to ensure that no more than a feasible amount of patients are recruited to the semistructured interview, the opt-in check box will be removed once enough participants have been recruited to achieve saturation of qualitative data themes.

The duration of the interview will be between 30 and 45 min. The interview will be recorded using a Dictaphone and transcribed verbatim for analysis.

##### Statistical Considerations

###### Sample Size

It is estimated that approximately 170 eligible cases will be seen across all sites over a 17-month recruitment period, with an approximate split of 50% functioning and nonfunctioning NET patients. With 80% consent rate and 10% attrition, this will provide a sample of 136 at baseline and 122 at 6-month follow-up. The expected sample will provide 80% power to detect medium-sized differences between groups on continuous outcomes (0.48 SD at baseline and 0.51 SD at follow-up), assuming a 2-sided, alpha=.05 independent samples *t* test.

###### Quantitative Analysis of Patient-Reported Outcome Measures, Demographics, and Clinical Data

Descriptive statistics will be used to summarize demographic and clinical characteristics and patient-reported outcomes by group at baseline and follow-up. Statistics will include counts and percentages for nominal valued variables and means and standard deviations or medians and interquartile ranges, as appropriate, for continuous valued variables.

Dichotomous valued variables will be compared between groups using Fisher exact test. Nominal valued variables will be compared between groups using Pearson chi-squared test of independence. Responses to patient-reported outcome measures at baseline and follow-up will be compared between groups using independent samples *t* tests; between-groups differences on EORTC QLQ-C30 scales and items will be interpreted using evidence-based guidelines [50]. In the absence of evidence-based guidelines for the SCNS and PROMIS measures, the Cohen d effect size will be calculated and interpreted using existing conventions [51]. If a nonparametric method is required, differences in medians will be examined using the bootstrap percentile method [52]. The number of bootstrap replications will be set at 10,000.

All data will be entered in SPSS version 23 or higher (Chicago, IL), and SPSS will be used for scoring, descriptive analysis, and parametric tests. If a nonparametric method is required, data will be imported into R version 3.3.3 (or higher), and the R package *pairwise CI* will be used for this purpose [53]. Alpha will be set at .05 for all analyses, and all tests will be 2-tailed.

###### Qualitative Analysis of Semistructured Interviews

Patient interviews will be transcribed and analyzed using qualitative content analysis. Specifically, interviews will be coded and categorized into 2 groups representing functioning and nonfunctioning NETs patient responses. Similar codes across the 2 categories for functioning and nonfunctioning NETs patients will then be explored as global themes informed by ground theory [54,55]. Coding labels will be created by 2 independent experienced qualitative researchers, who will examine and discuss each other’s coding and thematic analysis to promote rigor and interrater reliability [56].

#### Phase 2: Development of the Nurse-Led Phone-Based Intervention

In collaboration with the Unicorn Foundation, this study will develop and pilot test an intervention to augment this service.

##### Unicorn Foundation

The Unicorn Foundation is an Australian not-for-profit medical foundation that aims to educate and support people who have a NET, as well as their families, by promoting better awareness and knowledge of the disease. The Unicorn Foundation advocates research in the area and has worked with clinical researchers to raise money and fund pilot testing of NET clinical trials. In addition, the foundation independently funds the employment of a registered nurse, offering the support of a NET Nurse hotline.

Current utilization of the NET Nurse hotline has been estimated by the Unicorn Foundations data monitoring at approximately 350 incoming calls over a 6-month period, with callers originating across all Australian states and territories. In addition, the location of callers is estimated at 59% from metropolitan areas and 41% of callers from rural areas, where access to NET-specialized physicians is likely to be limited. In addition, the Unicorn Foundation website offers a range of online resources such as updates on current NET research, access to support groups, and information on NETs.

The accessibility to this service nationally makes it an optimal avenue for disseminating and implementing evidenced-based approaches to provide better quality of care for those accessing information. This study will expand upon this service and provide a more structured response for patients with information that is tailored to meet the needs of NET patients based on their clinical characteristics and from information that has been derived from both clinical and patient-reported outcome data collection.

##### Development Framework

In line with the Schofield and Chambers framework, the design and development of the intervention will be guided by the 7 key features required to achieve effective translation into usual care and practice [40]:

1. Targeting cancer type and stage: To gain an understanding of the problem, patients recruited to the study are classified as either functioning or nonfunctioning by their treating oncologist based on their clinical presentation of distinctive hormonal syndromes and symptoms associated with functioning NETs [9,11,19-21] versus the asymptomatic profile of nonfunctioning NETs [23]. Intervention content will be developed based on quantitative and qualitative data on patient experiences and care preferences. The intervention will target the 2 subgroups and provide tailored supportive care around aspects of information on their disease, symptom burden, and self-care management.
2. Tailoring to individual’s unique needs: The delivery of the intervention will be tailored to suit individual needs. NET patients utilizing the Unicorn Foundation website will have access to online information and tools to access support. Users will be triaged online via the website based on symptom severity and distress. Relevant information will be delivered online as part of the intervention content will be tailored in depth based on the first phase of triaging. If the patients’ needs or concerns are not resolved, the intervention approach will be progressively stepped up to the top tier, which will be a phone-based nurse follow-up. In adopting this approach, the nurse will be able to prioritize the most vulnerable patients, increasing efficacy of the service and making patient encounters more meaningful and useful.
3. Promoting self-management: Promoting self-management of disease by providing patients with the resources and skills to address issues around symptom assessment, problem solving, and goal setting may reduce distress and ultimately health care use by enhancing the uptake of health behaviors [50,51]. The intervention will provide users with online information and resources to help facilitate self-assessment and symptom management. Nurse-led phone-based follow-up will also use motivational interviewing techniques to enhance self-management behavior change by adopting a client-centered method thought to encourage self-motivation [52].
4. Efficient intervention delivery: The delivery method of this intervention utilizes existing resources and infrastructure via the Unicorn Foundations website and Nurse Hotline service. Using this method of delivery adopts a low-intensity approach, improving efficiency by being a tool that can be easily integrated as an ongoing clinical service.
5. Ensuring evidence-based and theoretical grounding: The content of the intervention will be based on theory and available evidence. Content development will be based on analysis of both quantitative and qualitative data collected in the prospective cohort study that will describe the psychosocial, QoL, and clinical characteristics of patients with functioning and nonfunctioning NETs. Patient experiences and care preferences reported during semistructured interviews will also form part of the deliverable content of the intervention. The delivery mode is likely to incorporate media visual, textual, and diagrammatic resources and one-to-one telephone support. For example, the intervention may include patient experience videos, flowchart diagrams of treatment pathways, and fact sheets.
6. Specifying protocol training and adherence: A standardized manual for the one-to-one nurse-led telephone consultations will be developed, specifying the (1) content of the intervention consisting of, but not limited to, a description of NETs and their subgroups, method of triaging patients, symptom burden, patient care preferences, assessment of needs, and coaching in relevant self-care strategies and (2) training and supervision procedures. The development of a protocol of standardized content and training will ensure a comprehensive knowledge base and consistent reproducible delivery of the intervention content.
7. Confirming stakeholder acceptability: Stakeholder acceptability will be optimized throughout the design process by involving consumers, consumer advocacy group, allied health professionals, medical oncologists, and clinical NET nurses in the development of the phone-based nurse-led intervention [53,57]. All members of the stakeholder committee will also review iterative revisions of the intervention and resource manuals.

#### Phase 3: Pilot/Feasibility Study

##### Study Setting

The study will be conducted via the Unicorn Foundation website.

##### Pilot Testing of Intervention

###### Pretesting the Intervention

Online triaging will be tested by the research team to ensure functionality. A nurse with experience managing NET patients will be trained in intervention delivery, including confirming eligibility of callers, assessing the online triage, and delivery of intervention material. The nurse will also be trained on eliciting and responding to emotional and informational cues, goal setting, and motivational interviewing and will receive feedback on practice sessions. All intervention content will be recorded for quality assurance.

Over a 12-week period, 20 callers will be recruited via the Unicorn Foundation Nurse Hotline and invited to participate in a small-scale pilot study. Callers will be screened to determine their eligibility to participate. Eligibility criteria will be the same as used in phase 1, with the addition of the participant being able to speak as well as understand English to ensure comprehension and effective communication via the Nurse Hotline. Once eligibility is confirmed, callers will be given access to the online triage tool, which will determine the intervention content that will be delivered. Participants will also have the option to receive a nurse-led phone-based follow-up to address any ongoing concerns.

###### Assessment of Successful Implementation

The success of the implementation of the intervention will be assessed based on the taxonomy of 8 conceptually distinct implementation outcomes. These consist of, acceptability, adoption, appropriateness, feasibility, fidelity, implementation cost, penetration, and sustainability [58,59]. Of these outcomes, 3 will be assessed to determine the successful implementation of the intervention. These consist of (1) acceptability and satisfaction with various aspects of the intervention; (2) appropriateness, relevance, and suitability of the intervention; and (3) feasibility, practicability, and suitability for everyday use of the intervention [58,59].

Measurement of the acceptability appropriateness and feasibility domains will consist of a semistructured interview. Both the participants and the provider will be questioned on their level of satisfaction with the content and its delivery as well as the usefulness and practicability of the material. The number of people invited to participate in the pilot study and the percentage who consent to participate will be recorded as a measure of feasibility and indication for suitability of everyday use.

Interviews will be transcribed, coded, and categorized into 2 groups representing functioning and nonfunctioning NETs patient categories. Similar codes across the 2 categories for functioning and nonfunctioning NETs patients will then be explored as global themes informed by ground theory [54,55]. Coding labels will be created by 2 independent experienced qualitative researchers, who will examine and discuss each other’s coding and thematic analysis to promote rigor and interrater reliability [56]. These analyses will be used to iteratively refine the intervention.

##### Data Management

###### Data Monitoring

The study has received ethics approval from the Human Research Ethics Committee of Peter MacCallum Cancer Centre. No significant risks to participants are anticipated. Questionnaire responses will be scored within 3 working days of being received, so as to ensure that any high distress scores reported in questionnaires will be reported to the treating clinician and/or nurse coordinator in a timely manner. Participants may then be referred to receive treatment from a qualified psychologist; however, this procedure will be determined by, and is dependent upon, each site’s standard of care with respect to referral procedures.

### Safety

Any adverse or unexpected outcomes that occur as a result of the study will be documented, and copies will be provided to site investigators and the principal investigator within 24 hours. The principal investigator will proceed to report any such adverse event to the Human Research Ethics Committee.

### Ethics and Dissemination

Ethics approval was obtained from the Human Research Ethics Committee of Peter MacCallum Cancer Centre (project number 16/08L), Human Research Ethics Committee of the Northern Sydney Local Health District in New South Wales (project number RESP/16/73), Human Research Ethics Committee of the Central Adelaide Local Health Network in South Australia (project number Q20160901), Human Research Ethics Committee of Royal Perth Hospital (project number RGS0000000632), and Human Research Ethics Committee of the Royal Brisbane and Women’s Hospital (project number 17/QRBW/400). Results will be widely disseminated to the funding body and oncology conferences and meetings and through peer-reviewed publications.

## Results

Currently, the project is progressing through phase 1 and has completed recruitment. A total of 138 participants have been recruited to the study. To date, we have patient-reported outcome data from 122 participants at baseline and 87 participants at 6-month follow-up. Of these, qualitative data have been collected from 35 participants who consented to a semistructured interview. Phase 2 and phase 3 of the project are yet to be completed.

## Discussion

Limited research suggests that patients with NETs have significantly impaired QoL compared with the general population [30,60]. However, given the heterogeneity of this diagnosis and the broad spectrum of symptom severity patients may experience [11,21], current research also fails to delineate how this clinical variability impacts patient supportive care needs. This study will address and improve on the availability of disease-specific information as well as informing the design of supportive care tailored for the unique needs of the NET patient population. Using the Schofield and Chambers framework [40], a tailored intervention will be developed that will provide targeted supportive care for patients with NETs according to the clinical characteristics and psychosocial profile of patients with functioning and nonfunctioning NETs.

This research initiative will be the first concerted effort to differentiate the priorities and needs of patients with functioning and nonfunctioning NETs comparing the QoL, psychological morbidity, health care system experiences, and clinical profile of these 2 distinct NET patient subgroups. This project is innovative, and to our knowledge, this study is a world-first initiative that will collaborate with the NET national advocacy group, consumers, behavioral scientists, medical oncologists, and specialist NET nurses to iteratively design and pilot test a phone-based nurse-led intervention. The expected benefits of this study are that in combining the expertise across disciplines to form collaboration between medical oncologists, professional nurses, behavioral scientists, consumers, and consumer advocacy groups, this initiative capitalizes on multidisciplinary perspectives to develop a model that will improve the supportive care of patients with NETs.

To date, no research has delineated the differences between patients with functioning and nonfunctioning NETs in terms of their experiences, QoL, psychosocial, and daily functioning needs. Therefore, the development of an intervention that targets these differences and can provide tailored supportive care that is accessible to all patients across metropolitan and rural areas has the propensity to optimize current care.

The intervention will build on the existing Unicorn Foundation online platform and phone counseling to improve the current care of patients with NETs by providing targeted tailored support via the Unicorn Foundation website and nurse-led phone-based support hotline. This intervention also has the potential to be accessible to all patients irrespective of their demographical location. This initiative offers a novel, patient-centered approach to the supportive care of patients diagnosed with NETs that will lead the way nationally and internationally. This work will lead to a large-scale randomized controlled trial to evaluate the impact of this novel intervention on patient health outcomes.

## Abbreviations

EORTC QLQ-C30: Euopean Organization for Research and Treatment core, Quality of Life Questionnaire
GINET21: Gastrointestinal Neuroendocrine QoL Module
NETs: neuroendocrine tumors
PICF: Participant Information and Consent Form
PROMIS: Patient-Reported Outcomes Measurement Information System
QoL: quality of life
SCNS: Supportive Care Needs Survey
